# Reanalyze unassigned reads in Sanger based metagenomic data using conserved gene adjacency

**DOI:** 10.1186/1471-2105-11-565

**Published:** 2010-11-18

**Authors:** Francis C Weng, Chien-Hao Su, Ming-Tsung Hsu, Tse-Yi Wang, Huai-Kuang Tsai, Daryi Wang

**Affiliations:** 1 Biodiversity Research Center, Academia Sinica, Taipei, 115, Taiwan; 2 Institute of Information Science, Academia Sinica, Taipei, 115, Taiwan; 3 Research Center for Information Technology Innovation, Academia Sinica, Taipei, 115, Taiwan; 4 Department of Computer Science and Information Engineering, National Taiwan University, Taipei, 106, Taiwan

## Abstract

**Background:**

Investigation of metagenomes provides greater insight into uncultured microbial communities. The improvement in sequencing technology, which yields a large amount of sequence data, has led to major breakthroughs in the field. However, at present, taxonomic binning tools for metagenomes discard 30-40% of Sanger sequencing data due to the stringency of BLAST cut-offs. In an attempt to provide a comprehensive overview of metagenomic data, we re-analyzed the discarded metagenomes by using less stringent cut-offs. Additionally, we introduced a new criterion, namely, the evolutionary conservation of adjacency between neighboring genes. To evaluate the feasibility of our approach, we re-analyzed discarded contigs and singletons from several environments with different levels of complexity. We also compared the consistency between our taxonomic binning and those reported in the original studies.

**Results:**

Among the discarded data, we found that 23.7 ± 3.9% of singletons and 14.1 ± 1.0% of contigs were assigned to taxa. The recovery rates for singletons were higher than those for contigs. The *Pearson *correlation coefficient revealed a high degree of similarity (0.94 ± 0.03 at the phylum rank and 0.80 ± 0.11 at the family rank) between the proposed taxonomic binning approach and those reported in original studies. In addition, an evaluation using simulated data demonstrated the reliability of the proposed approach.

**Conclusions:**

Our findings suggest that taking account of conserved neighboring gene adjacency improves taxonomic assignment when analyzing metagenomes using Sanger sequencing. In other words, utilizing the conserved gene order as a criterion will reduce the amount of data discarded when analyzing metagenomes.

## Background

The investigation of metagenomes, which sequences DNA from mixed environmental samples directly, has provided insights into microbial communities, and is now widely used to study various living microorganisms as a system [1-4]. The major goal of metagenomic studies is to determine the systemic properties of a microbial community, including the genetic, metabolic, ecological, physiological and behavioral aspects of all community members [5-8]. Some high-throughput pipelines have been constructed for high-performance computational analysis of metagenomic data [9,10]. The pipelines facilitate taxonomic binning of huge amounts of sequencing data by referring to databases of known microbial genomes [11-14]. Based on the above approaches, recent investigations have revealed enormous variations among the microbiomes of diverse environments, such as human intestinal and salivary microbiota [15-17], microbial communities growing on sunken whale skeletons [18], and open ocean communities [19,20].

To study genetic materials from natural environmental samples, Sanger sequencing technologies have been used for generating DNA sequences [15,16,20]. Yet, much more metagenomic datasets were conducted using next generation sequencing (NGS) technologies (e.g., Roche GS-FLX, Illumina 1G analyzer, and Applied Biosystems SOLiD) which yield shorter fragments ranging from 30 bp to 350 bp [21]. As huge amount of sequencing data were produced, analysis tools have become a critical player in data interpretation [22]. For example, when scaffolds and contigs are assigned to phylogenetically related groups, GLIMMER [23], GeneMark.hmm [24], and MetaGene [25] are widely used to identify putative coding sequences (CDSs). Subsequently, the taxonomic assignment of CDSs is performed using BLAST [26] or other homology search tools [27] with sequence databases. Recently, some advanced taxonomic assigning tools like MEGAN [28], Phymm [29], PhyloPythia [30] were published. However, the majority of the reads only contain partial coding regions. Thus, they were usually unidentified because of the limited match length. For example, in two distal gut microbiomes, approximately 40% of 139,521 high-quality reads were discarded after sequence assembly. Moreover, approximately 40% of 50,164 CDSs predicted by using the GLIMMER package were excluded from further analysis due to insignificant BLAST scores [15]. In 13 healthy Japanese individuals, 33% of 1,065,392 shotgun reads failed to assemble, and 25% of 662,548 CDSs (identified by MetaGene) were excluded from further analysis [16]. It is estimated that existing analytical methods discard approximately 30-40% of metagenomic data from Sanger approaches [11,15,16,18,19,31]. Considering the drawback, we were motivated to re-analyzed the discarded reads of metagenomes generated using Sanger sequencing.

**Figure 1:**
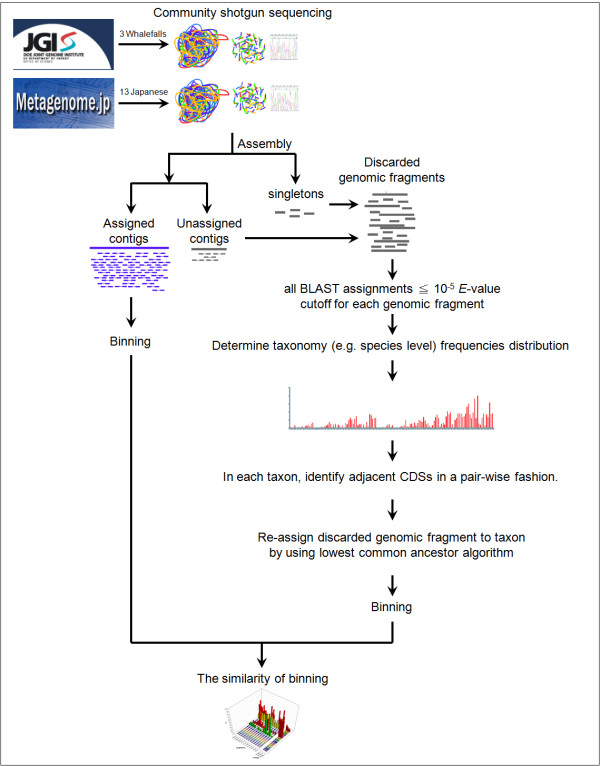
**Overview of the proposed approach**.

To overcome the limitations of current binning approaches, which rely heavily on the BLAST hit score, we propose a method for assigning reads discarded by the original studies (Figure 1). The new approach combines the BLAST search scores (two or more CDSs in a read) and the concept of conserved gene adjacency. The rationale is based on the theory that genomes are shuffled, so local gene-order conservation reflects the specificity of microbial organisms [32]. For example, the conservation of the gene order in prokaryotes is known to be an important feature; hence, it has been used in function inference [33,34]. Since gene order conservation is a genomic feature that is extensively conserved between closely related species [35,36], the trend should be universal in prokaryotic genomes [37]. Furthermore, it is known that overlapping gene pairs are frequently observed in microbial chromosomes [38] and conserved across species [39] in all three transcriptional directional classes: unidirectional (→→), convergent (→←), and divergent (←→) [40,41]. Therefore, we argue that, if a genomic fragment contains two or more adjacent CDSs that are identified by BLASTX, it is reasonable to assign the sequence by using the proposed strategy, which combines two BLASTX hit scores and the adjacency of the two genes.

A recent study showed that the average gene density in prokaryotic genomes is one gene per 1,000 nucleotides [41], which is close to the sequence length yielded by whole genome shotgun sequencing. Thus, we were aware that the read length would be the limitation of this approach. In our study, we only applied the analyses to conventional Sanger reads, which have higher potential to contain adjacent gene information than NGS. We first used simulated metagenomes to estimated the ratio of discarded singletons that may contain at least two neighboring genes [42]. We found that approximately 49% of discarded singletons contained gene pairs in all three transcriptional directional classes. Subsequently, we collected data from conventional metagenome projects that were generated via Sanger sequencing, and re-analyzed the fragments that were discarded from two types of metagenomic data, 13 healthy Japanese individuals [16] and the skeletons of whale carcasses (whale fall) [18]. Two types of genomic fragments, assembled contigs and raw single reads (singletons), were analyzed separately. The results showed that between 12.9% and 31.4% of the discarded data were assigned to taxa. Furthermore, the microbial compositions using discarded data and those reported in previous studies [15,16,18] were highly consistent in the family and phylum ranks. Therefore, we conclude that the proposed metagenomic sequencing approach provide a more comprehensive overview of the functional and taxonomic content of a microbiome.

## Results and Discussion

NGS technology facilitates the investigation of microbial communities. Because of the enormous number of short DNA fragments in metagenomic datasets, some bioinformatics tools, such as MEGAN [28], PhymmBL [29] and TACOA [43], have been developed for phylogenetic classification. However, current taxonomic binning methods have to discard a large number of sequences due to low homology scores. To address this problem, we developed a method that assigns discarded genomic fragments by combining the BLAST search scores and the criterion of gene adjacency. First, to assess the feasibility of our approach, we used simulated metagenomes to analyze the distribution of the number of CDSs in discarded singletons. In the simulated data sets, which had different levels of complexity (simLC, simMC and simHC, see Methods), we found that nearly half of the discarded singletons contained two or more partial CDSs (Table 1), suggesting that some of the discarded datasets could still be assigned to taxa.

**Table 1:** Number (and ratio) of discarded singletons that did not contain any CDS and those that contain one, two and three or more CDSs in the simulated metagenomes.

Number of CDS on singleton	simLC		simMC		simHC	
			
	Singletons	%	Singletons	%	Singletons	%
0	2575	6.2	2637	6.5	3986	6.0
1	18219	44.0	18072	44.8	29926	45.0
2	17549	42.4	16832	41.7	27874	41.9
3 or more	3081	7.4	2838	7.0	4738	7.1
Total singletons	41424	100	40379	100	66524	100

### Binning discarded metagenomic fragments

We used two kinds of metagenomes from whale fall samples (contigs) and healthy Japanese individuals (singletons) respectively (see Methods). Since the singletons were not available in the public domains, we repeated the assembly strategies and obtained similar datasets. As shown in Table 2, between 4,990 and 7,660 discarded contigs were collected from three whale fall microbiomes; and for the Japanese individuals, between 7,078 and 28,244 discarded singletons were collected after the assembly process. Under the proposed approach, between 12.9% and 14.9% of the discarded contigs in the whale fall samples were assigned to taxa. In the group of Japanese individuals, we were able to assign between 16.9% and 31.4% of the discarded singletons (see Table 3) to taxa. Based on the results, we suggest that the proposed binning strategy can be applied for re-analyzing the discarded reads of metagenomic data.

**Table 2:** Summary of collected metagenomic fragments.

Data type I (contigs)				Assigned	Unassigned	
					
	Location	Position	Total contigs	CDSs^a^	Contigs^b^	Average length (bp)
whale fall sub. 1	Pacific Ocean, Santa Cruz Basin (N33.30 W 119.22)	section of rib bone	35975	33139	7039	1167
whale fall sub. 2	Pacific Ocean, Santa Cruz Basin (N33.30 W 119.22)	bone	32459	32395	7660	1199
whale fall sub. 3	West Antarctic Peninsula Shelf (S65.10 W64.47)	bone	27130	26841	4990	1357

			**Our duplication**^**c**^			
			
**Data type II (singletons)**				**Assigned**	**Unassigned**	
				
	**Sex**	**Age**	**Total reads**	**CDSs**^**d**^	**Singletons**	**Average length (bp)**

Japanese In-A	Male	45 years	76434	29247	13399	1057
Japanese In-B	Male	6 months	80617	14718	7078	1058
Japanese In-D	Male	35 years	84237	48033	28244	1034
Japanese In-E	Male	3 months	80852	27860	10838	1124
Japanese In-M	Female	4 months	89340	26350	8456	1008
Japanese In-R	Female	24 years	85787	45438	21661	998
Japanese F1-S	Male	30 years	78452	40427	15378	1005
Japanese F1-T	Female	28 years	81348	46487	21780	958
Japanese F1-U	Female	7 months	82525	27332	11791	969
Japanese F2-V	Male	37 years	80772	49411	19733	1006
Japanese F2-W	Female	36 years	79163	42750	16961	1039
Japanese F2-X	Male	3 years	80858	41337	19351	1040
Japanese F2-Y	Female	1.5 years	79754	49315	20061	990

^a ^Genes with best hits at 30% identity or higher in Archaea and Bacteria kingdoms from JGI

^b ^Genes with best hits less than 30% identity in Archaea and Bacteria kingdoms from JGI

^c ^Phred and PCAP assembly package for Japanese samples.

^d ^The number of predicted open-reading frames showing similarity to genes in the "in-house NR database".

**Table 3:** Summary of reassignments using discarded metagenomic data.

Data type I (contigs)		Re-assigned				
				
	Contigs	Contigs	Average length (bp)	Rate (%)	r (phylum)	r (family)
whale fall sub. 1	7039	1050	1388	14.9	0.98	0.92
whale fall sub. 2	7660	995	1295	12.9	0.98	0.77
whale fall sub. 3	4990	720	1400	14.4	0.97	0.79

**Data type II (singletons)**		**Re-assigned**				
				
	**Singletons**	**Singletons**	**Average length (bp)**	**Rate (%)**	**r (phylum)**	**r (family)**

Japanese In-A	13399	3542	1074	26.4	0.95	0.85
Japanese In-B	7078	2050	1073	28.9	0.99	0.90
Japanese In-D	28244	5542	1061	16.9	0.89	0.72
Japanese In-E	10838	2888	1129	26.6	0.99	0.95
Japanese In-M	8546	2159	1057	25.2	0.93	0.86
Japanese In-R	21661	3993	1020	18.4	0.96	0.80
Japanese F1-S	15378	3216	1018	20.9	0.95	0.82
Japanese F1-T	21780	4395	971	20.1	0.93	0.59
Japanese F1-U	11791	3711	983	31.4	0.99	0.99
Japanese F2-V	19733	4007	1020	20.3	0.90	0.61
Japanese F2-W	16961	4011	1052	23.6	0.89	0.77
Japanese F2-X	19351	4402	1054	22.7	0.92	0.66
Japanese F2-Y	20061	4766	1002	23.7	0.96	0.82

The consistency between binning with discarded fragments and that in the original studies was tested by the Pearson correlation coefficient (r).

### The consistency of binning with discarded fragments compared to the strategies in previous studies

**Figure 2:**
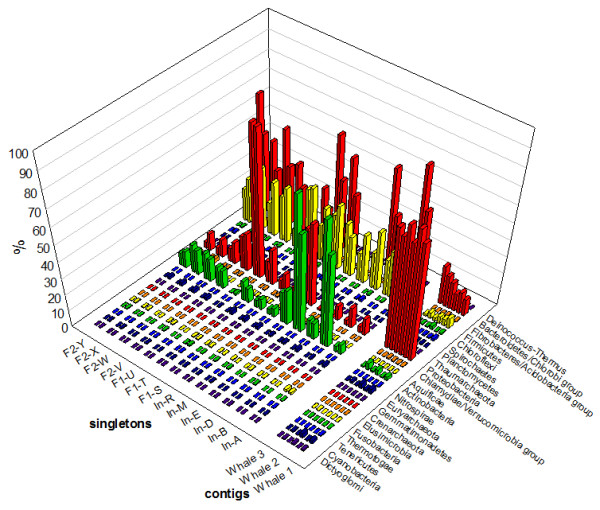
**Compositional View of 16 microbiomes in the phylum rank**. The bars depict the detailed contribution of microbiomes with 22 phyla represented on two types of genomic fragments, contigs and singletons. For each microbiome, the similarity of binning between our re-assignments and that of the original studies was compared. The consistency of the two datasets is represented by the *Pearson *correlation coefficient (Table 3).

To validate our approach, we compared the proposed taxonomic binning strategy using discarded datasets with the strategies in previous studies [15,16,18]. We used *Pearson *correlation coefficient to evaluate the similarity of the two groups. For taxonomic assignments using homology search tools, reads were assigned down to the class, order, family, and genus ranks [3,5,11,14,16,29,43]. Therefore, we separated the comparison into phylum and family ranks to describe the similarity between the original results and our binning results. We found that the results derived by our taxonomic binning strategy and those reported in previous studies were consistent. The correlation coefficients were 0.94 ± 0.03 in the phylum rank and 0.80 ± 0.11 in the family rank (Table 3). For example, the compositional view of Japanese individual F1-U showed a high degree of similarity between the two binnings (Figure 2). The correlation coefficient was 0.99 in both the phylum rank and the family rank. The consistency between the two datasets indicates that taxonomic binning using discarded data is as representative as the binning strategies used in previous studies.

To further evaluate our approach, we used 10,000 simulated singletons (simMC) for taxonomic binning to quantify the performance of our analysis. As shown in Table 4, the discarded singletons with the length of ~1 kb (Table 2) were correctly assigned with sensitivity between 36.8-25.9% and specificity between 93.3-79.0% between phylum and genus (using *E-*value 10^-2^, hits numbers 250). The hit number is positively correlated with the sensitivity but is negatively correlated with specificity, while the *E-*values do not seem to affect accuracy. In comparison with same method but without considering the gene adjacency, our approach showed a slight decrease in specificity but increased in sensitivity. For example, in family and genus ranking, the sensitivity is approximately four times higher than the method that does not consider gene adjacency (Table 4). Furthermore, because of the lack of similar analysis for discarded reads, here, we referred to previous studies using whole metagenomic data. For example, in TACOA [43], which reportedly performed better than PhyloPythia [44], the average sensitivity for binning 1 kb singletons ranged from 71% in the superkingdom rank to 22% in the class rank; and the average specificity ranged from 73% in the superkingdom rank to 64% in the class rank. Although our dataset sources (discarded dataset) were different from TACOA (whole dataset), the results indicate that with suitable filters and criteria, reliable information in the discarded data can be retrieved.

**Table 4:** Sensitivity and specificity of taxonomic binning at different taxonomic ranks using discarded dataset of simMC.

			Accuracy							
			
Criteria			P		O		F		G	
**adjacency**	***E*-value**	**hits**	**Sn**	**Sp**	**Sn**	**Sp**	**Sn**	**Sp**	**Sn**	**Sp**

with	1e-2	50	31.8	97.2	28.3	90.8	27.6	84.8	24.1	88.8
		
		150	34.9	94.5	30.8	86.2	30.0	79.3	25.3	82.0
		
		250	36.8	93.3	31.7	84.0	30.7	77.0	25.9	79.0
		
		350	37.9	92.7	32.0	83.0	30.7	75.8	25.7	77.6
	
	1e-4	50	29.7	97.4	27.8	91.1	27.4	85.1	23.7	89.2
		
		150	34.1	94.9	30.3	87.1	29.5	80.2	25.5	83.1
		
		250	36.0	93.7	31.4	84.9	30.3	77.8	25.7	80.2
		
		350	37.1	93.2	31.7	83.9	30.5	76.7	25.8	78.7
	
	1e-6	50	29.1	97.5	27.3	91.2	26.8	85.1	23.4	89.2
		
		150	33.1	94.9	29.7	87.1	28.8	80.2	24.7	83.1
		
		250	35.0	93.7	30.8	84.9	29.8	77.8	25.5	80.2
		
		350	36.2	93.2	31.3	83.9	30.2	76.7	25.8	78.7

without	1e-2	50	31.3	99.6	9.6	97.8	5.9	89.1	4.1	93.3
		
		150	20.0	99.7	7.3	94.0	5.2	88.1	4.5	92.0
		
		250	17.5	99.6	7.3	94.0	5.2	88.1	4.5	92.3
		
		350	16.3	99.5	7.3	94.0	5.2	88.1	4.5	92.3

Criteria considering gene adjacency and without considering gene adjacency were tested separately. P: phylum, O: order, F: family, G: genus. Sn and Sp denote sensitivity (%) and Specificity (%).

It has been observed that HGT (horizontal gene transfer) occurs frequently in prokaryotes [45]. Such a mechanism of genetic variability within a species may create bias in taxonomic binning based on a traditional homology search method. However, not all genes are equally itinerant, and they do not exhibit the same HGT behavior [46,47]. Preferential HGT correlates strongly with the functions of different types of genes. For example, informational genes (those involved in transcription, translation, and related processes) are far less likely to be transferred horizontally than operational genes (e.g. housekeeping functions) because they are complex systems [46]. In genome wide studies using 116 prokaryotes [48], the authors reported 46,759 HGT events in a total of 3,245,653 ORFs, but the horizontal transfer clusters (more than one gene) were relatively low (only 1,357 cases). Our approach considers the BLAST search scores and the criterion of conserved gene adjacency. Hence, the bias resulting from HGT should be relatively low compared to that of other approaches using a single hit.

## Conclusions

Since a large amount of metagenomic data generated using Sanger sequencing fails to satisfy the cut-off for taxonomic binning, we introduce a criterion based on a genomic feature, namely, the conservation of gene adjacency between prokaryotes. Our analysis suggests that considering the conserved neighboring gene adjacency reduces the amount of data discarded by current methods. In fact, a latest update of MEGAN software has incorporated similar analysis for pair reads, and the assignment for LCA-gene has been improved considering the conserved adjacency [49]. In addition, we are aware that the vast majority of recent metagenomic datasets were produced by NGS technologies (e.g., Roche GS-FLX, Illumina 1G analyzer, and Applied Biosystems SOLiD), and our analysis can only be applied to datasets with longer reads, such as Sanger. Yet, Roche's first-generation instrument, 454 GS 20 (released in 2005), yielded 100-bp reads, the latest version GS Junior System (released in 2009, Roche) already yielded demonstrably higher read lengths, exceeding 500 bp. Hopefully, the limitations of sequence length will be resolved in the near future, and our study will provide a basis for analyzing metagenomic data.

## Methods

### Collection of metagenomes and microbial genome sequence

Figure 1 shows an overview of our methodology. We used two kinds of metagenome samples: sunken whale skeletons (whale fall) and human distal guts. Three independent whale fall samples were collected in 2005 [18]. The assembled sequence data was downloaded from NCBI ftp://ftp.ncbi.nih.gov/genbank/wgs/ with accession numbers AAFY01000001-AAFY01028151 (whale fall 1), AAFZ01000001-AAFZ01029934 (whale fall 2), and AAGA01000001-AAGA01026232 (whale fall 3). The microbiomes of distal guts were collected from 13 healthy Japanese individuals (six individuals and members of two unrelated families) [16]. The data was downloaded from the Human Metagenome Consortium, Japan (HMGJ, http://www.metagenome.jp/). Table 2 summarizes the metagenomic fragments that we collected.

To obtain information about gene adjacency, we downloaded microbial genomes from the NCBI ENTREZ Genome Project database http://www.ncbi.nlm.nih.gov/genomes/lproks.cgi. A total of 3,072,893 protein sequences were obtained from 939 complete microbial genomes and 576 plasmids in August 2009. The sequences had to be processed by formatdb before they could be used by the BLAST program.

### Collection of discarded genomic fragments

We analyzed two types of discarded genomic fragments: contigs that failed to meet the criteria in the original studies and singletons that were left for analysis. The discarded contigs, which were obtained from the DOE Joint Genome Institute (JGI, http://www.jgi.doe.gov/), contained genes that failed to pass the 30% BLAST identity cut-off, or they had no hits in the Archaea and Bacteria kingdoms of each microbiome.

To collect the discarded singletons, we followed the assembly strategy described in Kurokawa K et al. [16]. For the 13 Japanese samples, the original trace archives (chromatogram files) were downloaded from the DNA Data Bank of Japan (DDBJ, http://www.ddbj.nig.ac.jp/). To read the DNA sequence chromatogram files, we adopted the Phred program [50,51], which is widely used for base-calling and characterizing the quality of DNA sequences. Finally, the shotgun reads from the 13 samples were assembled using the PCAP software [52] with the default parameters. The number and average length of the remaining singletons from the Japanese individuals are shown in Table 2. The slight differences between our statistics and those reported in previous studies may be due to different parameter settings.

### Collection of simulated datasets

To estimate the proportion of discarded singletons that contain at least two genes from real metagenomes, we downloaded three simulated metagenomic data sets of varying complexity as benchmarks and calculated the number of CDSs in each singleton. The three simulated datasets, a low-complexity community (simLC), a moderate-complexity community (simMC) and a high-complexity community (simHC), were compiled by combining sequencing reads randomly selected from 113 genomes [42]. After assembling the simulated datasets using Phrap (v3.57), all remaining singletons were published by the Department of Energy (DOE) Joint Genome Institute. They are available through the Integrated Microbial Genome (IMG) system. In addition, we also used simMC to evaluate the performance of our taxonomic assignment method. In total, there are 15,197 contigs and 40,379 singletons that Phrap assembler failed to assemble. We randomly selected 10,000 non-redundant singletons from simMC for analysis.

### Taxonomic assignment of discarded genomic fragments

To incorporate the conservation of gene order into the taxonomic classification, each discarded genomic fragment was screened for protein encoding genes via a BLASTX search against the NCBI ENTREZ Genome Project database. An expected cut-off value (*E*) of 10^-5 ^was used to select the top 250 potential coding elements as the default settings. (We discuss the selection criteria in **Accuracy evaluation using simulated datasets**).

Normally, the best hits are selected from BLAST results, but best hits do not provide information on adjacent genes. Therefore, the top 250 hits were selected instead. In our strategies, adjacent gene pair is a pair of genes that are directly next to each other in a given chromosome. Thus, each hit was grouped with its corresponding species. These hits were then compared in a pair-wise fashion in order to identify adjacent CDSs. The transcriptional direction (unidirectional (→→), convergent (→←), and divergent (←→)) of all identified adjacent CDSs should be consistent with the genomic arrangement of reference genomes. Next, the pairs with inconsistent genomic arrangement were removed. Subsequently, among the remaining pairs, we ran the lowest common ancestor (LCA) algorithm used in MEGAN [28] to analyze the data. The source code is provided in the supplementary material (see [Additional file 1]). It requires perl and Basic Local Alignment Search Tool (BLAST) on the work station. The program has been tested by using several resources listed in **Collection of discarded genomic fragments **and in **Collection of simulated datasets**.

### Comparison of binning discarded fragments in the proposed approach and the original studies

To assess the consistency of binning results using the discarded dataset and the binning results reported in original studies, we compared the quantitative contribution of microorganisms in discarded data set and original data set. Contigs and singletons were performed separately. Because the phylogenetic taxonomies constructed by the NR database (used in previous studies) and the NCBI ENTREZ Genome Project database (used in our study) were not consistent, we selected 22 phyla and 166 families that were consistent in both databases to estimate the similarity of the binning results (see [Additional file 2]). To quantify the similarity, we calculated *Pearson *correlation coefficient. We found that, in each environment, the taxonomic binning was dominated by a limited number of phylotypes; and the remaining phylotypes only made a small contribution. To avoid over-estimation resulting from the latter, all phylotypes less than five were combined before calculating *Pearson *correlation coefficient in both datasets.

### Accuracy evaluation using simulated datasets

The selection of appropriate criteria may have a critical effect on our system's performance. In Table 4, the relationships between the criteria (*E*-value threshold (10^-2^, 10^-4^, and 10^-6^) and BLAST hits numbers (50, 150, 250 and 350)) and the accuracy of our system were evaluated using a simulated discarded dataset. Twelve combinations (3 *E*-values * 4 BLAST hits numbers) were tested for the performance evaluation.

Taxonomic reassignment for simulated data was evaluated by comparing the assignments made by our method to those of the real corresponding taxa in different taxonomic ranks (i.e., species, genus, family, order, class, phylum and superkingdom). In this study, we employed the adapted definition of sensitivity and specificity [43,53]. The accuracy was evaluated for each taxonomic class. Let the *i*-th taxonomic class of taxonomic rank *r *be denoted as class *i*. The true positives (*TP_i_*) are defined as the number of genomic fragments correctly assigned to class *i*; the false positives (*FP_i_*) are defined as the number of fragments from any class *j *≠ *i *that is wrongly assigned as *i*. The false negatives (*FN_i_*) are defined as the number of fragments from class *i *that is erroneously assigned to any other class *j *≠ *i*. For a genomic fragment whose taxonomic class cannot be inferred, the algorithm classifies it as "unclassified". The unclassified (*U_i_*) are the numbers of fragments from class *i *that cannot be assigned to a taxonomic class.

The sensitivity (**Sn**_*i*_) for a taxonomic class *i *is defined as the percentage of fragments from class *i *correctly classified. It is computed by:

$${\text{Sn}}_{i}=\frac{TP_{i}}{TP_{i}+FN_{i}+U_{i}}$$

The reliability (expressed in percentage) of the predictions made by the classifier for class *i *is denoted as specificity (**Sp**_*i*_). It is measured using the following equation:

$${\text{Sp}}_{i}=\frac{TP_{i}}{TP_{i}+FP_{i}}$$

To select appropriate *E-*value threshold, the data in Table 4 were examined. Since the results indicated that the *E-*values do not affect the performance of taxonomic binning, we selected a loose criterion (*E-*value 10^-5^) as default. The hit number is positively correlated with the sensitivity but is negatively correlated with specificity, (Table 4), the hit number 250 was selected as default considering the sensitivity, specificity and also the run-time required.

## Authors' contributions

DW and HKT formulated the studies, participated in the experiment design, and drafted the manuscript. FCHW participated in the program design of the study and helped draft the manuscript. CHS, MTH and TYW collected the data and performed the statistical analysis. All the authors read and approved the final manuscript.

## Supplementary Material

Additional file 1 **Reassignment_using_gene_adjacency.pl**. Perl script for reassignment using gene adjacency. Click here for file

Additional file 2 **Supplemental Table S1**. The phylotypes used to estimate the binning similarity. Click here for file
