# Supplementary material for: Reanalyze unassigned reads in Sanger based metagenomic data using conserved gene adjacency
Source: BMC Bioinformatics. 2010 Nov 18;11:565. doi: 10.1186/1471-2105-11-565 (PMC3098102; doi:10.1186/1471-2105-11-565)
Supplement: Additional file 2 — Supplemental Table S1. The phylotypes used to estimate the binning similarity. [file 1471-2105-11-565-S2.DOCX]

**Supplemental Table S1.** The phylotypes used to estimate the binning similarity. 22 phyla and 166 families.

| Phylum |  | Family |
| --- | --- | --- |
| Actinobacteria |  | Acidimicrobiaceae, Acidothermaceae, Actinosynnemataceae, Beutenbergiaceae, |
|  |  | Bifidobacteriaceae, Catenulisporaceae, Coriobacteriaceae, Corynebacteriaceae, |
|  |  | Dermabacteraceae, Dermacoccaceae, Frankiaceae, Jonesiaceae, Kineosporiaceae, |
|  |  | Microbacteriaceae, Micrococcaceae, Micromonosporaceae, Mycobacteriaceae, |
|  |  | Nocardiaceae, Nocardioidaceae, Nocardiopsaceae, Propionibacteriaceae, |
|  |  | Pseudonocardiaceae, Rubrobacteraceae, Streptomycetaceae |
| Aquificae |  | Aquificaceae, Hydrogenothermaceae |
| Bacteroidetes/Chlorobi group |  | Bacteroidaceae, Chlorobiaceae, Flavobacteriaceae, Flexibacteraceae, |
|  |  | Porphyromonadaceae, Rhodothermaceae, Sphingobacteriaceae |
| Chlamydiae/Verrucomicrobia |  | Chlamydiaceae, Opitutaceae, Parachlamydiaceae, Verrucomicrobiaceae |
| group |  |  |
| Chloroflexi |  | Chloroflexaceae, Herpetosiphonaceae, Thermomicrobiaceae |
| Crenarchaeota |  | Desulfurococcaceae, Pyrodictiaceae, Sulfolobaceae, Thermofilaceae, Thermoproteaceae |
| Cyanobacteria |  | Nostocaceae, Prochlorococcaceae |
| Deinococcus-Thermus |  | Deinococcaceae, Thermaceae |
| Dictyoglomi |  | Dictyoglomaceae |
| Elusimicrobia |  | Elusimicrobiaceae |
| Euryarchaeota |  | Archaeoglobaceae, Halobacteriaceae, Methanobacteriaceae, Methanocaldococcaceae, |
|  |  | Methanococcaceae, Methanocorpusculaceae, Methanomicrobiaceae, Methanopyraceae, |
|  |  | Methanosaetaceae, Methanosarcinaceae, Methanospirillaceae, Picrophilaceae, |
|  |  | Thermococcaceae, Thermoplasmataceae |
| Fibrobacteres/Acidobacteria |  | Acidobacteriaceae, Solibacteraceae |
| group |  |  |
| Firmicutes |  | Bacillaceae, Clostridiaceae, Clostridiales Family XI. Incertae Sedis, Clostridiales Family |
|  |  | XVIII. Incertae Sedis, Enterococcaceae, Eubacteriaceae, Halanaerobiaceae, |
|  |  | Heliobacteriaceae, Lactobacillaceae, Leuconostocaceae, Listeriaceae, Natranaerobiaceae, |
|  |  | Paenibacillaceae, Peptococcaceae, Planococcaceae, Staphylococcaceae, Streptococcaceae, |
|  |  | Syntrophomonadaceae, Thermoanaerobacteraceae, Thermoanaerobacterales Family III. |
|  |  | Incertae Sedis, Thermodesulfobiaceae |
| Fusobacteria |  | Fusobacteriaceae |
| Gemmatimonadetes |  | Gemmatimonadaceae |
| Nitrospirae |  | Nitrospiraceae |
| Planctomycetes |  | Planctomycetaceae |
| Proteobacteria |  | Acetobacteraceae, Acidithiobacillaceae, Aeromonadaceae, Alcaligenaceae, |
|  |  | Alcanivoracaceae, Alteromonadaceae, Anaplasmataceae, Bartonellaceae, |
|  |  | Bdellovibrionaceae, Beijerinckiaceae, Bradyrhizobiaceae, Brucellaceae, Burkholderiaceae, |
|  |  | Campylobacteraceae, Cardiobacteriaceae, Caulobacteraceae, Chromatiaceae, |
|  |  | Colwelliaceae, Comamonadaceae, Coxiellaceae, Desulfobacteraceae, Desulfobulbaceae, |
|  |  | Desulfomicrobiaceae, Desulfovibrionaceae, Ectothiorhodospiraceae, Enterobacteriaceae, |
|  |  | Erythrobacteraceae, Francisellaceae, Geobacteraceae, Hahellaceae, Halomonadaceae, |
|  |  | Helicobacteraceae, Hydrogenophilaceae, Hyphomonadaceae, Idiomarinaceae, |
|  |  | Legionellaceae, Methylobacteriaceae, Methylococcaceae, Methylophilaceae, |
|  |  | Moraxellaceae, Myxococcaceae, Nautiliaceae, Neisseriaceae, Nitrosomonadaceae, |
|  |  | Oceanospirillaceae, Oxalobacteraceae, Pasteurellaceae, Pelobacteraceae, |
|  |  | Phyllobacteriaceae, Piscirickettsiaceae, Polyangiaceae, Pseudoalteromonadaceae, |
|  |  | Pseudomonadaceae, Psychromonadaceae, Rhizobiaceae, Rhodobacteraceae, |
|  |  | Rhodocyclaceae, Rhodospirillaceae, Rickettsiaceae, Shewanellaceae, |
|  |  | Sphingomonadaceae, Syntrophaceae, Syntrophobacteraceae, Vibrionaceae, |
|  |  | Xanthobacteraceae, Xanthomonadaceae |
| Spirochaetes |  | Brachyspiraceae, Leptospiraceae, Spirochaetaceae |
| Tenericutes |  | Acholeplasmataceae, Entomoplasmataceae, Mycoplasmataceae |
| Thaumarchaeota |  | Nitrosopumilaceae |
| Thermotogae |  | Thermotogaceae |
